# Hyperreflective Dots in Central Fovea Visualized by a Novel Application of Visible-Light Optical Coherence Tomography

**DOI:** 10.1155/2024/5823455

**Published:** 2024-07-09

**Authors:** Michael A. Krause, Marta Grannonico, Brooke P. Tyler, David A. Miller, Weijia Fan, Mingna Liu, Roman V. Kuranov, Hao F. Zhang, Xiaorong Liu, Peter A. Netland

**Affiliations:** ^1^ Department of Ophthalmology University of Virginia, Charlottesville, Virginia, USA; ^2^ Department of Biology University of Virginia, Charlottesville, Virginia, USA; ^3^ Department of Biomedical Engineering Northwestern University, Evanston, Illinois, USA; ^4^ Department of Psychology University of Virginia, Charlottesville, Virginia, USA

## Abstract

Visible-light optical coherence tomography (vis-OCT) is a novel noninvasive retinal imaging system that offers improved resolution compared to conventional near-infrared (NIR) OCT systems. Here, we utilized vis-OCT to produce fibergrams (vis-OCTF) for the first time in human patients, enabling *en face* visualization and precise quantification of hyperreflective dots in the central fovea in two patients. We also directly compare the imaging qualities of conventional vis-OCT and NIR-OCT. Vis-OCT generated a 3 × 3 mm^2^*en face* image with an impressive axial resolution of 1.3 *μ*m, whereas NIR-OCT produced an *en face* image with a larger field of view (FOV) (9 × 9 mm^2^) but a lower resolution of 7.0 *μ*m. Moreover, vis-OCTF unveiled clear images of hyperreflective dots in the fovea of both patients, which were not discernible in the NIR-OCT *en face* images. Foveal dots have often been linked to several age-related and pathological conditions. The high-resolution images generated by vis-OCTF enable more precise characterization of changes in retinal sublayers within the central fovea.

## 1. Introduction

Optical coherence tomography (OCT) is a noninvasive imaging technique widely used in ophthalmology to visualize retinal structures [1]. Clinical near-infrared (NIR) OCT systems operate at wavelengths from 830 to 1550 nm, which limits axial resolution. For example, the Spectralis OCT (Heidelberg Engineering, USA) has a lateral resolution of 14 *μ*m and an axial resolution of 7.0 *μ*m [2]. Such parameters inherently limit the visualization of subtle changes in retinal layers and sublayers. Visible-light OCT (vis-OCT), which operates at wavelengths from 510 to 610 nm [3], provides higher resolution due to higher optical scattering by tissue in the visible-light range compared to NIR [4, 5]. Our previous studies showed that vis-OCT provides 1.3-*μ*m axial resolution in the retina, which is more than a 5-fold improvement over the 7.0-*μ*m axial resolution of the clinical NIR-OCT. Vis-OCT has previously been used to analyze individual sublayers of the IPL in patients with and without glaucoma [6].

To directly assess the performance of NIR-OCT compared to vis-OCT and vis-OCT fibergraphy (vis-OCTF), we acquired vis-OCT images from the same patients immediately following NIR-OCT imaging. The vis-OCT volume was used to generate vis-OCTF, an *en face* visualization of the retinal nerve fiber layer (RNFL). Previous studies in mice and tree shrews show that we can characterize individual RGC axon bundles within the RNFL in vivo [7, 8], but to our knowledge, this technique has not been utilized in humans. Vis-OCTF images showed clearly identifiable hyperreflective dots [9] in the central fovea of two patients. Interestingly, these hyperreflective dots could be identified in both NIR-OCT and vis-OCT B-scan images, but not on NIR-OCT *en face* images. A side-by-side comparison revealed that vis-OCT offered substantially improved image quality versus NIR-OCT.

## 2. Case Presentations

Case 1 was a 79-year-old Caucasian female with nuclear sclerotic cataracts and intermediate age-related macular degeneration (AMD). The past medical history was unremarkable. Snellen's best-corrected visual acuity (BCVA) on presentation was 20/25 and 20/60. Intraocular pressures (IOPs) were 11 and 14 mmHg by Goldmann applanation. Slit lamp examination (SLE) was notable for nuclear sclerotic cataracts in both eyes and multiple hard drusen in the macula of both eyes.

Case 2 was a 74-year-old Caucasian female with ocular hypertension in both eyes and suspected glaucoma. The past medical history was unremarkable. Snellen's BCVA was 20/20 in both eyes. IOPs were 18 mmHg in both eyes by Goldmann applanation. SLE was notable for nuclear sclerotic cataracts in both eyes and large optic nerves with an intact neuroretinal rim.

## 3. Imaging Methods

Both patients were imaged with commercial NIR-OCT and vis-OCT. The commercial NIR-OCT system used was OCT Spectralis® (Heidelberg Engineering, USA) with 30° of field of view (FOV) which corresponds to a 9 × 9 mm^2^*en face* image. The system provides 14 *μ*m of lateral resolution and 7.0 *μ*m of axial resolution, with a 40 kHz A-line rate. Each volume took approximately 8.8 s to acquire.

The vis-OCT system used was the Aurora X2 vis-OCT system (Opticent Inc., Evanston, IL). The vis-OCT system is a noninvasive imaging technology that generates a 3 × 3 mm^2^*en face* image with 7.0 *μ*m of lateral resolution and 1.3 *μ*m of axial resolution, with a 40 kHz A-line rate. The incident power was set below 250 *μ*W on the cornea. The laser used in Aurora X2 has been certified by the Food and Drug Administration as a nonsignificant-risk device for laser safety.

Before each imaging session, vis-OCT irradiation power was measured using a calibrated power meter (PM100D; Thorlabs, Newton, NJ, USA) [6]. From the patient's standpoint, the imaging process is very similar to NIR-OCT. However, the image acquisition time is longer for vis-OCT, and the brightness of the light source can be bothersome to some patients. For each patient, we acquired one vis-OCT volume (512 A-lines/B-scan, 512 B-scans/volume) from the same eye with the fovea aligned in the center of the FOV. Each OCT volume was acquired in 7.6 s. The scan covered a volume of 3 × 3 × 1.2 mm^3^ in the retina. We adjusted the optical focus and the reference arm path length for each FOV to maximize image quality.

**Figure 1:**
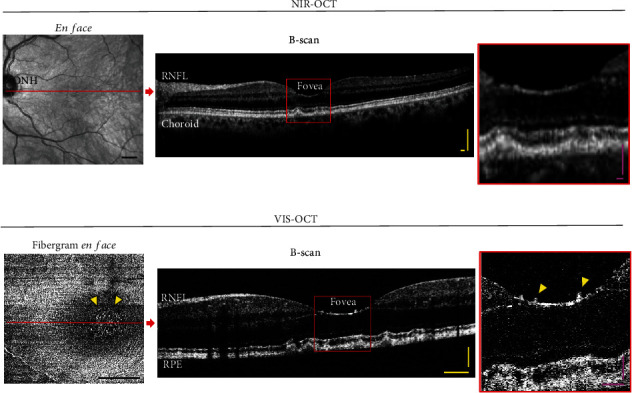
Vis-OCT offers higher resolution than NIR-OCT. Patient #1 (intermediate AMD) was imaged the same day with a commercial (a) NIR-OCT (Spectralis) and (b) Vis-OCT (Aurora X2). (a) NIR-OCT en face shows retina vascularization in the FOV. B-scan image reconstructed along the red line. Magnified view of the region highlighted by the box. (b) Vis-OCTF (fibergram *en face*) shows individual axon bundles and hyperreflective dots (yellow arrowheads) around the fovea. A speckle-reduced B-scan image was reconstructed along the red line. Magnified view of the region highlighted by the red box. ONH: optic nerve head; RNFL: retinal nerve fiber layer; RPE: retinal pigment epithelium. Scale bars: black = 1 mm; yellow = 100 *μ*m; pink = 50 *μ*m.

**Figure 2:**
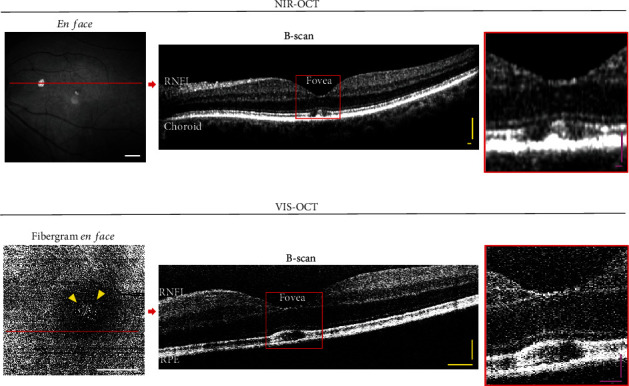
Hyperreflective dots in the central fovea were clearly visualized by vis-OCTF. Patient #2 (suspect glaucoma) was imaged on the same day with (a) NIR-OCT and (b) vis-OCT. (a) NIR-OCT *en face* shows retina vascularization in the FOV. B-scan image reconstructed along the red line. Magnified view of the region highlighted by the box. (b) Vis-OCTF (fibergram *en face*) shows individual axon bundles and hyperreflective dots (yellow arrowheads) around the fovea. A speckle-reduced B-scan image was reconstructed along the red line. Magnified view of the region highlighted by the red box. ONH: optic nerve head; RNFL: retinal nerve fiber layer; RPE: retinal pigment epithelium. Scale bars: white = 1 mm; yellow = 100 *μ*m; pink = 50 *μ*m.

Vis-OCT fibergrams were generated from each vis-OCT volume (Figures 1 and 2) [7, 8]. We used an intensity-based threshold method to detect the surface of the retina. For each B-scan, the segment between the internal limiting membrane (ILM) and NFL/GCL boundaries was automatically cropped [10, 11]. The mean intensity projection between the two boundaries was used to generate the fibergram image, which is composed of RGC axon bundles and surrounding vasculature. Segmentation errors were manually corrected. In addition, we averaged five B-scans along the *x*-axis and plotted an 11-pixel-thick (~15 *μ*m) arc to produce a speckle-reduced B-scan at selected locations across the foveal region [6]. Note that the generation of a NIR-OCT fibergram is not possible because (1) the Spectralis OCT dataset only provides limited B-scans/volume (~19 B-scans/volume) [12] which is not sufficient to recreate a fibergram *en face* image and (2) the NIR-OCT axial resolution is not high enough to accurately resolve individual axon bundles [13].

**Figure 3:**
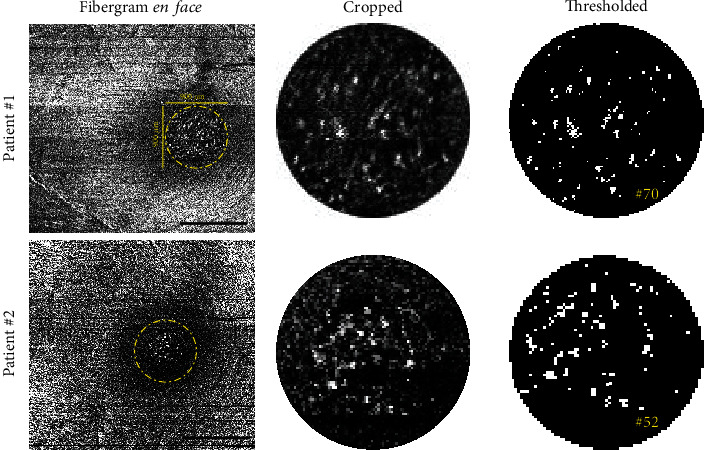
Presentation of the 900 × 900 *μ*m cropped *en face* fibergram images. Cropped vis-OCTF *en face* images from both patients illustrate the presence of the hyperreflective dots within the foveal area (yellow dashed line circle). Cropped images were exported to ImageJ, thresholded, and then analyzed to determine the number of hyperreflective dots. Black scale bars = 1 mm.

To quantify hyperreflective dots in the vis-OCT fibergram, we applied the previous methodology used by Corradetti et al. [9]. Briefly, we localized and cropped the foveal zone on the fibergram *en face* (~900 × 900 *μ*m, Figure 3). The obtained cropped image was exported in ImageJ, binarized, and thresholded to quantify the hyperreflective dots. Patient 1 had 70 hyperreflective dots, and Patient 2 had 52 hyperreflective dots.

## 4. Discussion

In this study, Patient #1 (intermediate AMD) and Patient #2 (glaucoma suspect) were imaged on both commercial NIR-OCT (Figures 1(a) and 2(a)) and vis-OCT with vis-OCTF (Figures 1(b) and 2(b)). The vis-OCTF images clearly demonstrate the hyperreflective dots in the central fovea observed on the B-scans from both NIR-OCT and vis-OCT. Corradetti et al. have also previously demonstrated hyperreflective dots in *en face* NIR-OCT images. However, this required manual segmentation of the *en face* image [9]. Notably, vis-OCTF allowed accurate characterization and quantification of the hyperreflective dots compared to the corresponding vis-OCT B-scan. We quantified the number of hyperreflective dots in the vis-OCT fibergram and found a similar number of hyperreflective dots in the fovea in both patients. Our results agreed with the age-matched data presented by Corradetti et al. [9].

Before widespread use of OCT, Yokotsuka, Kishi, and Shimizu first identified what was termed white dot fovea by using scanning laser ophthalmoscopy and electron microscopy [14]. Hyperreflective dots in the fovea can be associated with various age-related and pathological conditions. For example, one study showed that the number of hyperreflective dots in the fovea increases with age, particularly after the age of 50 [9]. In addition to age, various factors can influence the presence and characteristics of hyperreflective dots in the fovea. Structural changes in the retina associated with disease-induced inflammation and stress are thought to contribute to the formation of these dots [15]. One specific condition linked to hyperreflective dots is AMD, a progressive retinal disease that affects the macula and can lead to central vision loss. Studies have shown that AMD is associated with changes in hyperreflective dots, suggesting their potential role in the pathogenesis of the disease [15]. On the other hand, diabetic macular edema (DME) and retinal vein occlusion (RVO) do not seem to induce similar changes in hyperreflective dots [15]. More studies are also needed to elucidate the pathophysiologic relevance of the number and size of hyperreflective spots and the underlying mechanisms in disease conditions. Given our findings in these two patients, it is possible that hyperreflective dots are underrecognized. In conclusion, vis-OCTF introduces unique imaging capabilities not achievable with conventional NIR-OCT, which will enable more precise analysis of changes in retinal sublayers.

## Data Availability

All data generated or analyzed during this study are included in this article. Requests for further information can be made to the corresponding authors.
